# 

**Published:** 1859-12

**Authors:** 


### CONTENTS.

"Dental Periodical Literature."—"Dental Journals." ..... 251
Professional Health ..... 253
The D. M. C.'s Reply to "One of Her Boys." ..... 256
Introductory Address. By C. B. CHAPMAN ..... 259
Hunter on Slayton ..... 274
Thoughts for Dentists. By Dr. W. A. PEASE ..... 277
Correspondence ..... 283
Michigan Dental Association ..... 285
Indiana State Dental Association ..... 286
Selections ..... 287
Physiology and Pathology of the Nervous System. By Dr. BROWN-SEQUARD ..... 287
Amber Bases for Artificial Teeth ..... 290
New Discovery ..... 292
Discovery of the Anæsthetic Effects of Chloroform ..... 293
Editorial ..... 294
Another Local Society ..... 294
Sander's Compound ..... 294
Decision of the "Hard Rubber" Contest ..... 295
A Practical Treatise on Operative Dentistry ..... 295

